# A review of hedgehog signaling in cranial bone development

**DOI:** 10.3389/fphys.2013.00061

**Published:** 2013-04-02

**Authors:** Angel Pan, Le Chang, Alan Nguyen, Aaron W. James

**Affiliations:** Department of Pathology and Laboratory Medicine, David Geffen School of Medicine, University of CaliforniaLos Angeles, Los Angeles, CA, USA

**Keywords:** craniofacial abnormalities, Sonic Hedgehog (SHH), Indian Hedgehog (IHH), cranial suture patterning/signaling, calvarial bone differentiation

## Abstract

During craniofacial development, the Hedgehog (HH) signaling pathway is essential for mesodermal tissue patterning and differentiation. The HH family consists of three protein ligands: Sonic Hedgehog (SHH), Indian Hedgehog (IHH), and Desert Hedgehog (DHH), of which two are expressed in the craniofacial complex (IHH and SHH). Dysregulations in HH signaling are well documented to result in a wide range of craniofacial abnormalities, including holoprosencephaly (HPE), hypotelorism, and cleft lip/palate. Furthermore, mutations in HH effectors, co-receptors, and ciliary proteins result in skeletal and craniofacial deformities. Cranial suture morphogenesis is a delicate developmental process that requires control of cell commitment, proliferation and differentiation. This review focuses on both what is known and what remains unknown regarding HH signaling in cranial suture morphogenesis and intramembranous ossification. As demonstrated from murine studies, expression of both SHH and IHH is critical to the formation and fusion of the cranial sutures and calvarial ossification. SHH expression has been observed in the cranial suture mesenchyme and its precise function is not fully defined, although some postulate SHH to delay cranial suture fusion. IHH expression is mainly found on the osteogenic fronts of the calvarial bones, and functions to induce cell proliferation and differentiation. Unfortunately, neonatal lethality of IHH deficient mice precludes a detailed examination of their postnatal calvarial phenotype. In summary, a number of basic questions are yet to be answered regarding domains of expression, developmental role, and functional overlap of HH morphogens in the calvaria. Nevertheless, SHH and IHH ligands are integral to cranial suture development and regulation of calvarial ossification. When HH signaling goes awry, the resultant suite of morphologic abnormalities highlights the important roles of HH signaling in cranial development.

## Introduction

Craniofacial morphogenesis, an intricate developmental process, begins with the synchronized development of head primordia, which involves several organizing centers located in the neural ectoderm, axial mesendoderm, and the cranial neural crest. The differentiation and spatial patterning of these tissues must occur before they can be successfully integrated (Hu and Helms, 1999). The Sonic Hedgehog morphogen (SHH) is one of the signals involved in the axial and dorsoventral definition of craniofacial and limb development (Chiang et al., 1996; Capdevila and Johnson, 2000; Anderson et al., 2012). Dysregulation of the Hedgehog (HH) signaling pathway results in a wide array of craniofacial defects including holoprosencephaly (HPE), hypotelorism, and cyclopia, amongst others (Belloni et al., 1996). Both Indian Hedgehog (IHH) and SHH have been well studied in cartilage and bone patterning throughout the axial, appendicular and facial skeleton (Hammerschmidt et al., 1997; Capdevila and Johnson, 2000; Chai and Maxson, 2006). In contrast, the role of HH signaling in calvarial ossification and cranial suture morphogenesis is a relatively new and less examined area of scientific investigation.

The embryonic development of the cranium and cranial suture complex begins as far back as neural crest cell migration, a process starting on murine embryonic day 8 (E8) and completed within 2 days (Slavkin, 1979). In humans, this period lies approximately between E19 and E38 (Dixon et al., 1997). After migration of the neural crest, the calvarial mesenchyme originates from both the paraxial mesoderm and the migrated cranial neural crest (Noden, 1983). The demarcations between the neural crest and mesoderm-derived bone have undergone evolutionary changes resulting in species-specific differences. In avian skulls this boundary has been found on the caudal border of the frontal bone and that a portion of the calvarium was derived from neural crest cells (Noden, 1975; Le Lievre, 1978). Later studies have proposed that avian skull may even arise entirely from neural crest cells (Couly et al., 1993). Murine studies, using Wnt1-Cre (a reporter for neural crest origin) mice, suggest that the entire frontal bone is derived from neural crest origin, while other skull bones principally originate from the paraxial mesoderm (Chai et al., 2000; Jiang et al., 2000, 2002; Brault et al., 2001; Chai and Maxson, 2006; Ishii et al., 2012). Nevertheless, the process of cranial skeletogenesis occurs in concert with the expansion of the brain. Neural crest cells originate from the dorsal neural tube and as the cells migrate they are pulled by the expansion of the epidermis to form the frontonasal process. The frontal primordium mesenchyme then forms the frontal bones, which differentiates into the primary bone plates (Iseki et al., 1995; Ting et al., 2009). Subsequent growth of the bone plates occurs until they meet, resulting in formation of a suture.

Characterized as regions of fibrous tissue between cranial bones, sutures function as intramembranous osteogenic sites, permit cranium growth, inhibit bone separation, and absorb shock (Baer, 1954; Cohen, 1993; Wilkie, 1997; Lenton et al., 2005). Cranial sutures are distinguished with respect to their adjacent cranial bones (i.e., the metopic suture lies between frontal bones, etc.). It is crucial that sutures remain in an undifferentiated and proliferative state as the brain develops while permitting the growth of new bone at the suture margins until the they fuse (Opperman, 2000).

Cranial suture fusion is a process whereby the cells in the middle of the suture complex mature into osteoblasts that eventually fuse into midline sutures (i.e., the sagittal and metopic sutures), which join end-to-end. In humans all sutures close around the end of adolescence (Madeline and Elster, 1995). In contrast, in mice, the transversely situated sutures (lamboid and coronal sutures), do not undergo ossification and remain overlapping and patent. During the stages before physiologic suture fusion, an imbalance between cell proliferation and osteoblast differentiation can cause inappropriate suture fusion, or craniosynostosis. Suture synostosis prevents further bone formation and accommodation of neurocranial growth, leading to craniofacial dysmorphology and central nervous system (CNS) impairments. Potential CNS effects of craniosynostosis include elevated intracranial pressure (Bristol et al., 2004; Hayward and Gonsalez, 2005), high incidence of learning disabilities (Kapp-Simon, 1998; Panchal et al., 2001; Magge et al., 2002), and impaired eyesight (Macintosh et al., 2012). Therefore, regulation of cranial suture morphogenesis is crucial for maintenance of suture patency and proper physical and cognitive development.

Growth factor regulation is one of the main mechanisms that coordinate calvarial patterning and ossification. The most commonly studied signaling pathways regulating suture fusion include Fibroblast Growth Factor (FGF), Transforming Growth Factor (TGF-β), and Bone Morphogenetic Protein (BMP) signaling (Longaker, 2001; Warren et al., 2003; Nie et al., 2006a,b; Rawlins and Opperman, 2008). The dura mater (or outer layer of meninges) secretes a variety of growth factors in order to regulate cranial suture fusion and suture patency in a paracrine fashion (Opperman et al., 1993; Bradley et al., 1997; Cooper et al., 2012). FGF2 demonstrates a significant mitogenic effect and thus may stimulate proliferation of osteoprogenitors in the dura mater and overlying suture mesenchyme (Spector et al., 2002; Li et al., 2007). As a result, a majority of syndromic synostoses arise from Fibroblast growth factor receptor (FGFR) mutations (Cohen, 2009). Additionally, TGF-βs also serves to initiate suture fusion as there are high levels of TGF-β1 and 2 expression in fusing as compared to patent cranial sutures (Opperman et al., 1997, 1999; Ko et al., 2009; Slater et al., 2009). Studies dysregulating TGF-β signaling have also been shown to significantly alter suture ossification and fate (fusion vs. patency) (Derynck et al., 1994; Rawlins and Opperman, 2008; Chim et al., 2011). While the periosteum lies in close proximity in the suture, it does not contribute significantly to the regulation of suture fusion (Opperman et al., 1994, 1995). The significance of BMP signaling in cranial suture fusions was made clear by studies involving the BMP antagonist Noggin (Warren et al., 2003). While BMP is expressed in fusing sutures, the Noggin expression that it induces is counteracted by FGF2 signaling. In comparison to FGF, TGF-β, and BMP signaling, the role of HH signaling in cranial suture morphogenesis is a relatively new focus of investigation, yet one of clear importance.

This review will first provide an overview of HH signaling, including normal signal transduction and pertinent regulators of HH signaling. Next, a comprehensive review of the known functions of HH signaling in the cranial suture complex is presented. In addition, the role of HH in endochondral ossification and cranial base formation is examined. Finally, a discussion of what remains unknown or unclear regarding HH signaling in cranial suture biology is presented.

## An overview of hedgehog signaling

The activity of the HH pathway was first identified in Drosophila, and its expression was later found in all vertebrates (Fietz et al., 1994). Three homologues of the Drosophila HH protein exist in vertebrates: SHH, IHH, and Desert hedgehog (DHH). DHH expression is primarily limited to the male reproductive tract, and the majority of DHH^−/−^ mice do not exhibit mutant phenotypes in most tissues (Parmantier et al., 1999; Yao et al., 2002; Cohen, 2003; Kawai et al., 2010). In contrast, SHH and IHH are essential to embryonic development, as either SHH- or IHH-deficient mice demonstrate multiple severe congenital anomalies and neonatal lethality that will be further discussed below (Chiang et al., 1996; St-Jacques et al., 1999; Hayhurst and McConnell, 2003). SHH plays a diverse and key role in the development of the head process, notochord, ventrolateral midbrain, and ventral forebrain. SHH also plays an indispensible function in limb development, including limb budding, anterior-posterior patterning of limb skeleton and regulation of right/left asymmetry (Capdevila and Johnson, 2000). In regards to the craniomaxillofacial skeleton, SHH in the forebrain mediates the development of mid and upper face, frontonasal and maxillary processes (Byrnes et al., 2009). Closely related to SHH by a gene duplication event (Schlosser and Wagner, 2004), IHH regulates chondrocyte differentiation and stimulates endochondral bone formation (Cohen, 2003). IHH stimulates the proliferation of chondrocytes at the growth plate and further in development, osteoblast differentiation; in addition, it also regulates chondrocyte hypertrophic differentiation through a negative feedback loop involving Ihh-parathyroid hormone-related protein (PTHrP) (Bitgood and McMahon, 1995; St-Jacques et al., 1999; Long et al., 2004). As well, IHH promotes ossification and fusion of the cranial and palatine bones, discussed further below (Lenton et al., 2011; Levi et al., 2011). Both SHH and IHH are found to be crucial regulators of osteogenesis and therefore of importance in cranial suture biology.

**Figure 1:**
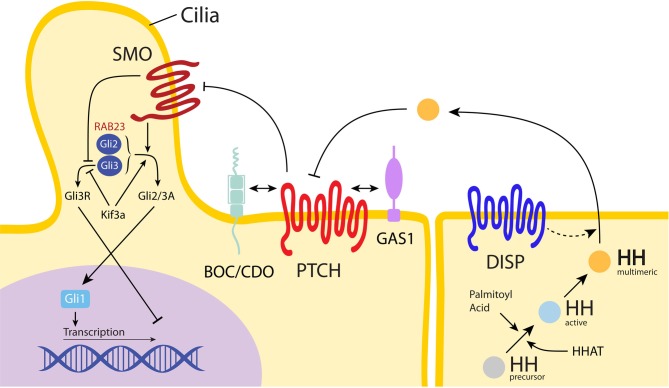
**Hedgehog pathway.** The Hedgehog (HH) ligand precursor undergoes a series of modifications until reaching an active, multimeric form (shown in yellow). All three HH ligands then signal through the same pathway: following multimeric HH ligand release from Dispatched (DISP) and secretion from the signaling cell, the HH ligand binds to Patched (PTCH) on the receiving cell, releasing Smoothened (SMO) from constitutive inhibition (Cohen, 2003). Released SMO then shuttles through the cilia. This signals activation of the Gli2/3 complex (shown as Gli2/3A), which promotes gene expression via Gli1, while simultaneously inhibiting the Gli3 repressor form (shown as Gli3R). In the presence of HH activated SMO, the Kif3a motor complex promotes Gli2/3A expression and inhibits repression by Gli3R (Rohatgi and Scott, 2008). HHAT is necessary for post-translational palmitoylation of HH; in the absence of HHAT, HH secretion is decreased (Dennis et al., 2012). In the presence of HH, BOC, CDO, and GAS1 bind to PTCH to form complexes which repress SMO, allowing downstream signaling through Gli to continue (Izzi et al., 2011). RAB23 (shown in red) functions as a negative regulator of the HH pathway, most likely through interaction with the Gli2 activator and Gli3 repressor forms. Please note, the depiction of SMO as a 7-transmembrane protein and both PTCH and DISP as a 12-transmembrane protein correspond to their respective biological conformations (De Rivoyre et al., 2006; Cohen, 2010).

All of the HH homologues undergo the same highly conserved HH signaling pathway that occurs through a three-step process. First, the insoluble HH morphogen is converted to a multimeric form, which renders it soluble and available for diffusion across cell membranes. This HH ligand is subsequently made active as HH ligands precursors undergo autocatalytic cleavage to form 19 kD proteins with a C-terminal cholesterol moiety (Porter et al., 1996; Cohen, 2003) (Figure 1). Palmitoyl acid then modifies the N-terminus, forming a palmitate (Pepinsky et al., 1998). After these two covalent lipid modifications, the HH ligand is now active and has an increased affinity for the cell membrane (Simpson et al., 2009). Second, a large transmembrane protein, Dispatched, releases the now lipid-anchored protein HH from the signaling cell, which allows HH binding to the receptor Patched (PTCH), a transcription inhibitor. After the HH ligand binds to PTCH, Smoothened (SMO), another transmembrane protein for downstream signaling, is freed from PTCH repression and transduces the signal intracellularly. In vertebrates, SMO leads to the transcription of target genes downstream through interaction with glioblastoma gene products (Gli) family of transcription factors (Gli1, Gli2, and Gli3). Recently, the so-called “non-canonical HH signaling pathway” has been discovered, which utilizes Gli independent pathways (Jenkins, 2009). This novel HH signaling has been studied during angiogenesis as well as cancer biology. However, this non-canonical signaling has not been sufficiently characterized for its operation in sutures, and will not be further addressed here (Chinchilla et al., 2010; Spek et al., 2010).

The Gli transcription factors are homologues of Cubitus interruptus (Ci), a transcription factor mediating HH signaling found in Drosophila (Huangfu and Anderson, 2006). Both Gli and Ci function similarly and act as key regulators of targeted gene expression. Gli proteins have been found to function as transcriptional activators, transcriptional repressors or both. Experiments have demonstrated that Gli2 and Gli3 both contain an amino-terminal repressor domain and a carboxyl-terminal activator domain that flanks the zinc fingers (Ruiz i Altaba, 1999; Sasaki et al., 1999; Wong and Reiter, 2008); however, Gli1 does not contain the repressor domain and so cannot be proteolytically processed (Ruiz i Altaba, 1998; Dai et al., 1999). Gene analysis has shown that Gli1 functions solely as a strong transcriptional activator (Hynes et al., 1997; Lee et al., 1997; Karlstrom et al., 2003). In mouse development, Gli1 does not appear to be essential since Gli1^−/−^ mutants are viable and survive from birth to adulthood and exhibit a normal phenotype (Park et al., 2000; Bai et al., 2002). In contrast, the other members of the Gli family, Gli2, and Gli3, are required for organ patterning, such as lung development and spinal cord patterning (Brewster et al., 2000; Bai et al., 2004; Rutter et al., 2010). Both Gli2 and Gli3 can act as both activators and repressors. Their bifunctionality is determined by the presence of HH signaling. When there are high concentrations of HH ligands, proteolytic processing of Gli2 and Gli3 is inhibited, which allows for their activator function. Without HH presence, these transcription factors may undergo cleavage to become repressors (Wang et al., 2000; Bai et al., 2004; Pan et al., 2006). The deviations of the carboxyl-terminal amino acid sequences of Gli2 and Gli3 have been found to result in differential processing (Pan and Wang, 2007). Gli2 proteins have inefficient protein processing and therefore remain transcriptionally active *in vivo* (Fuccillo et al., 2006). In contrast, the majority of Gli3 proteins are partially degraded, thus mainly functioning as a transcription repressor (Theil et al., 1999; Persson et al., 2002; Rallu et al., 2002). In summary, the Gli protein family controls HH signaling through transcription, both in the cranial suture region and throughout the organism.

## Craniofacial dysmorphism caused by hedgehog signaling

In the absence of an active HH ligand or interference with HH signal transduction, a transcriptional repression of HH target genes results in a slew of craniofacial anomalies (see Table 1). Aberrations in Gli3 are known to cause craniofacial dysmorphisms in both human and mice models. One result of altered Gli3 sequence is Greig cephalopolysyndactly syndrome, which causes metopic synostosis and is characterized by polydactyly and hypertelorism (Hui and Joyner, 1993; Quinn et al., 2012; Veistinen et al., 2012). Another caused by mutations in the Gli3 effector is Pallister-Hall Syndrome, with common craniofacial findings including disrupted midline development and abnormalities such as a short nose with flat nasal bridge, and cleft palate (Kuo et al., 1999; Naruse et al., 2010). In fact, the integral role of Gli3 as a transcriptional repressor is evident in studies with Gli3 null mice in which excessive osteoblastic proliferation and differentiation result in craniosynostotic phenotypes (Shimoyama et al., 2007). Interestingly, local application of recombinant FGF2 rescues loss of Gli3 as it stabilizes the increased osteoblastic proliferation observed in Gli3 deficient mice (Rice et al., 2010). HH signaling can also be effected by changes in the physical environment that transduces the signals of proteins in the HH pathway.

**Table 1:** **Genetic disorders in the hedgehog signaling network**.

**Syndrome name**	**Mutated gene**	**MIM**	Function of proteins^*^	Skeletal phenotypes^*^
Greig cephalopolysyndactyly syndrome	Gli3	175700	Transcriptional repressor of HH signaling	Pre- and post-axial polydactyly and syndactyly of hands and feet, slight hypertelorism, high prominent forehead
Pallister-hall syndrome	Gli3	146510	Transcriptional repressor of HH signaling	Disrupted midline development and craniofacial abnormalities including a short nose with flat nasal bridge and cleft palate
NBCCS	PTCH	109400	SHH receptor, inhibits SMO expression	Basal cell carcinomas, macroencephaly, cleft lip/palate, intracranial ectopic calcifications and facial dysmorphisms
Carpenter's syndrome	RAB23	201000	Negative regulator of SHH	Syndactyly, brachydactyly with shortening or absence of middle phalanges, craniosynostosis of midline and coronal sutures
Acrocapitofemoral dysplasia	IHH	607778	HH ligand	Clinically short stature with short limbs, brachydactyly, shortening or loss of middle phalanges
Holoprosencephaly 1	GAS1	236100	Co-receptor of PTCH	Missing phalanges and anterior digit syndactyly
Holoprosencephaly 11	CDO	614226	Co-receptor of PTCH	Lack of maxillary inscisors, primary palate, hypoplasia of the cartilage of the nasal septum
–	BOC	–	Co-receptor of PTCH	No abnormalities are observed in BOC^−/−^ mutants alone. However, BOC and CDO mutants exhibit severe craniofacial midline abnormalities such as elongated nose, cleft lip, and hypotelorism.
Asphyxiating thoracic dystrophy 3, Short ribpolydactyly syndrome, type II and type III	DYNC2H1	613091, 263520, 263510	IFT Protein, Ciliogenesis, signal transduction of HH pathway	Short rib polydactyly phenotype, shortened long bones, a narrow rib cage and polydactyly, variable malformations including cleft lip/palate
Asphyxiating thoracic dystrophy 5, Cranioectodermal dysplasia 4	IFT144	614376, 614378	IFT Protein, Ciliogenesis, signal transduction of HH pathway	Polydactyly, truncated ribs, craniosynostosis, exencephaly, reduced palatine bones and misshapen maxillary bones
–	HHAT	206500, 202650	Post-translational palmitoylation of HH proteins	Midface hypoplasia, agenesis of the jaw, loss of skeletal central bones, apoptosis in craniofacial mesenchyme

* Data from (Temtamy, 1966; Robinson et al., 1985; Shanley et al., 1994; Wild et al., 1997; Kuo et al., 1999; Lee et al., 2001; Liu et al., 2001, 2002; Cole and Krauss, 2003; Hellemans et al., 2003; Johnston et al., 2005; Zhang et al., 2006, 2011; Lo Muzio, 2008; Keaton et al., 2010; Naruse et al., 2010; Ashe et al., 2012; Dennis et al., 2012).

Abbreviations: GAS, growth arrest specific; HH, hedgehog; HHAT, hedgehog acyltransferase; IFT, intraflagellar transport; IHH, indian hedgehog; NBCCS, nevoid basal cell carcinoma syndrome; PTCH, patched; SHH, sonic hedgehog; SMO, smoothened.

Primary cilia serve an important and increasingly understood role in suture biology, as HH transduction initiates at the primary cilia (Tukachinsky et al., 2010). Studies involving cilial defects have shown that primary cilia are crucial to HH signaling. Currently, it is suggested that primary cilia provide an environment that facilitates interactions amongst the different pathway components in HH transduction (Ruat et al., 2012). During HH signaling, intraflagellar transport proteins (IFT), which are required for the production and preservation of cilia, have been found to affect the signal transduction of the HH pathway (Huangfu et al., 2003; Keady et al., 2012; Yang and Wang, 2012). IFT particles are formed by two complexes that use the Kif3 motor complex and the retrograde dynein motors to selectively import or export proteins between the cilium and cytoplasm (Ruat et al., 2012). Signal dependent transfer of PTCH, SMO, and Gli proteins requires ciliary transport in order to activate the HH pathway (Keady et al., 2012). Studies suggest that following PTCH regulation of SMO, SMO is consequently translocated to the cilium through the use of IFT proteins and interacts with Gli to promote Gli activation. Gli activators then move down the cilium to enter the nucleus and promote HH targeted genes (Huangfu and Anderson, 2006; Singla and Reiter, 2006). Interestingly, mutations that disrupt IFT proteins show phenotypes characteristic of SHH signaling defects. This observation extends to the craniofacial skeleton. For example, mutations in the IFT protein DYNC2H1, causes short rib polydactyly syndrome, a lethal autosomal recessive condition that features cerebral and skeletal abnormalities, such as HPE, in addition to other appendicular malformations (Dagoneau et al., 2009; Merrill et al., 2009; El Hokayem et al., 2012). Mutations in another IFT protein, IFT144, also result in craniofacial anomalies such as craniosynostosis and exencephaly, which results from deficient ciliogenesis and diminished response to upstream activation of HH signaling (Ashe et al., 2012). Gene analysis has found that IFT motor proteins such as Kif3a (Kinesin-like protein) are required for signaling of Gli transcription factors (Haycraft et al., 2005; Huangfu and Anderson, 2005; Liu et al., 2005). Kif3a conditional knockouts (Kif3a Wnt1-Cre) show a phenotype with severe cranial dysmorphisms including abnormal openings in the skull vault, associated with displacement of the neuroectodermal domains of SHH signaling (Brugmann et al., 2010). Interestingly, Kif3a deficient mouse skulls exhibit abnormalities partially overlapping with the SHH and IHH null phenotypes (see discussion below) (Koyama et al., 2007). In summary, cilia are involved in the regulation of HH signal transduction, although the precise mechanisms of this relationship are only partially elucidated.

Another potential cause of craniofacial abnormalities includes exposure to teratogens, which interfere with the HH signaling pathway. One of the main plant alkaloids that produce these deformities is the jervine family of alkaloids. Cyclopian deformities from alkaloids were first observed in the offspring of pregnant sheep grazing on *Veratrum Californicum*. Further experimentation found that these chemical compounds also trigger clefting and HPE in sheep and other animals by inhibiting the response of target tissues to HH ligands (Binns et al., 1963). The structural similarity of these alkaloids to cholesterol allows them to inhibit cholesterol's stimulatory effect on HH signaling (Cooper et al., 1998; Chen et al., 2002). For example, the steroidal alkaloid Cyclopamine (11-deoxojervine) causes teratogenic effects through direct binding of cyclopamine to the SMO heptahelical bundle thereby creating a PTCH independent pathway, thus resulting in HH pathway inhibition (Incardona et al., 2000; Chen et al., 2002). Cyclopamine is one of the causes of embryonic deficiency of midline and lower medial nasal prominence tissue, resulting in severe cranial defects including lateral cleft lip, cleft palate, and the cyclopia phenotype in many animal models including zebrafish and mice (Lipinski et al., 2010; Buttner et al., 2012).

Genetic mutations, rather than exposure to jervines, represent the major source of HH signaling abnormalities causing congenital dysmorphisms in humans. Currently, one third of all birth defects are craniofacial abnormalities, with HPE being the most common developmental disorder of the forebrain (Gorlin et al., 1990; Ming and Muenke, 1998; Ming et al., 1998). Despite the rare live-birth prevalence of 1 in 10,000 HPE infants, it may be as common as 1 in 250 conceptuses by some estimates (Vaz et al., 2012). In fact, SHH was the first gene identified to cause HPE in mice and humans from nonsense mutations or deletions that result in loss of function (Odent et al., 1999). In clinical studies, familial forms of HPE involve SHH gene mutations in up to 23% of affected families; much greater than the percentage of non-syndromic mutations, 1% (Roessler et al., 1997; Ming et al., 1998). SHH gene mutations are also responsible for lip and palatal defects as SHH signaling pathways regulate the epithelium and mesenchyme interactions that promote cell proliferation and palatal growth (Murray and Schutte, 2004). Additionally, IHH gene mutations, such as additional copies of the IHH locus, are associated malformations that result in syndactyly and craniosynostosis (Klopocki et al., 2011). Mutations in IHH can also cause acrocapitofermoral dysplasia, an autosomal recessive skeletal dysplasia resulting in shortening or loss of the middle phalanges (Gao et al., 2001; Hellemans et al., 2003; Byrnes et al., 2009).

Other gene mutations that induce HPE and result in HH pathway dysregulation involve PTCH, Growth arrest-specific 1 (GAS1), CDO, and BOC (Roessler and Muenke, 1998; Seppala et al., 2007; Allen et al., 2011; Bae et al., 2011; Zhang et al., 2012). CDO and BOC, the external binding domains of SHH, GAS1, the co-receptor of PTCH, and PTCH directly interact to activate the Gli transcription pathway (Izzi et al., 2011). GAS 1 has been found to promote SHH signaling in embryological development while being negatively regulated in response to SHH signaling (Allen et al., 2011). GAS 1 mutant mice have demonstrated various phenotypes similar to reduced SHH signaling (Liu et al., 2002). Co-existing CDO and GAS1 mutations in mice have resulted in HPE, therefore demonstrating these two co-receptors are essential to proper cranial patterning (Bae et al., 2011). Likewise, CDO and BOC double mutants also exhibit the HPE phenotype as well as extreme neural patterning defects. Mutations in BOC alone result in viable offspring with no HPE. Studies suggest that this absence may be compensated for by CDO, indicating that the two have similar functions (Zhang et al., 2012). Also, hedgehog acyltransferase (HHAT) loss of function decreases HH secretion and leads to the HPE phenotype; HHAT is necessary for modification of HH proteins (Chen et al., 2004; Dennis et al., 2012; Hardy and Resh, 2012). Yet another genetic mutation in the HH pathway is found in the mutation of the PTCH chromosome located on 9q22.3–q31 (Farndon et al., 1992). The loss of normal PTCH function leads to increased HH signaling and is thought to result in Nevoid Basal Cell Carcinoma Syndrome (NBCCS). This phenotype is characterized by basal cell carcinomas, macroencephaly, cleft lip and palate, intracranial ectopic calcifications, and facial dysmorphisms (Evans et al., 1993; Shanley et al., 1994; Lo Muzio, 2008; Zhang et al., 2011). Genetic mutations in regulator genes of HH signaling may also cause calvarial dysmorphisms. A mutation in the RAB23 gene leads to a dysfunctional repressor of HH signaling. This results in Carpenter's Syndrome, characterized by fusion of the midline sutures, obesity and syndactyly (Eggenschwiler et al., 2001; Jenkins et al., 2007). In summary, a substantial number of known human craniofacial anomalies in the HPE spectrum arise from HH and related genes mutations. See Table 1 for a summary of syndromes, gene mutations, and resultant craniofacial dysmorphisms.

## Role of hedgehog signaling in cranial suture morphogenesis

**Figure 2:**
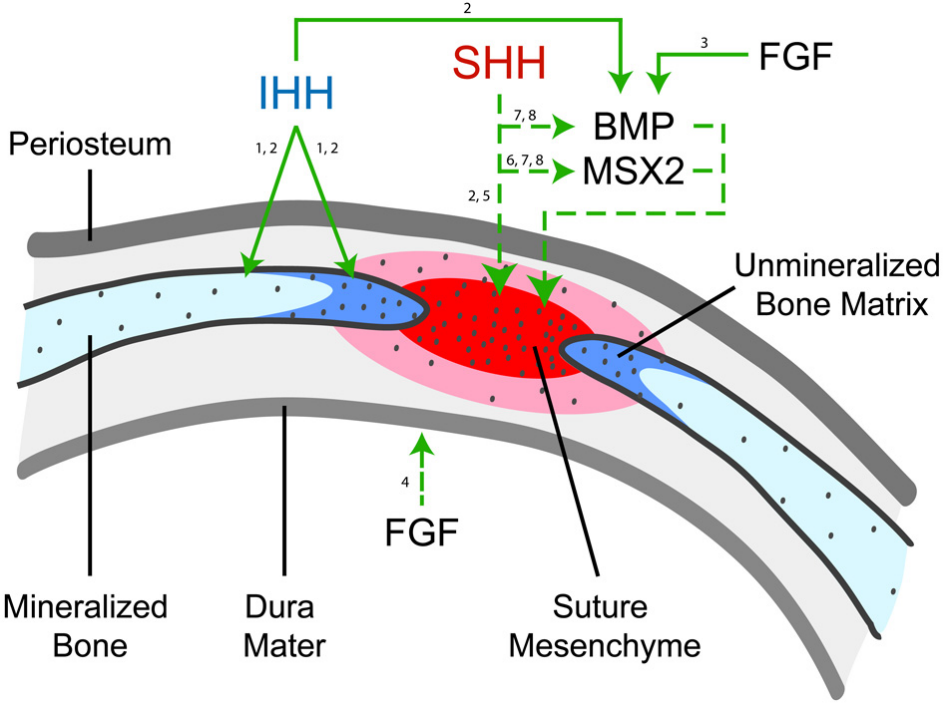
**HH Ligand expression and function in cranial suture morphogenesis.** Indian Hedgehog (IHH) is observed in the cranial bones (light blue), primarily at the osteogenic front (dark blue) (1, Jacob et al., 2007; 2, Lenton et al., 2011). In this figure, solid arrows represent consistent observations while dashed arrows represent postulated pathways. Studies have shown that IHH functions to increase new bone formation at the osteogenic fronts, likely through its upregulation of BMP2 and BMP4 (2, Lenton et al., 2011). Fibroblast growth factor (FGF) has been shown to promote BMP2/4 (3, Sahar et al., 2005) and is highly expressed in dura matter and is one of the main diffusible growth factors inducing sutural fusion (4, Li et al., 2007). In contrast, SHH has been observed to be expressed in a patched pattern in the midline suture mesenchyme (shown in red), although some disagreement regarding its expression pattern exists (2, Lenton et al., 2011). The function of SHH is less clear, although it has been postulated to function in maintaining suture patency (5, Kim et al., 1998). SHH may increase mesenchymal proliferation and suture mesenchyme thickness via promotion of MSX2 (6, Alappat et al., 2003), and similarities are present between the expression of SHH, MSX2, and BMP expression during neonatal craniofacial suture development (7, Liem et al., 2000; 8, Santagati and Rijli, 2003).

Both SHH and IHH ligands have demonstrated importance in cranial suture morphogenesis. Studies have identified discrete expression patterns of SHH vs. IHH ligands in the developing skull, which are distinct and indicate probable differences in function (Figure 2). Murine studies have shown that SHH gene expression in the skull occurs at the end of embryonic development (from E18 and onwards) in a discontinuous pattern in the osteogenic fronts of the midline suture mesenchyme, but is absent in the coronal sutures (Kim et al., 1998). However, other investigators found contradicting evidence of weak SHH expression in the parietal bones of E16.5 mice, as well as absent SHH expression in the midline suture mesenchyme (Lenton et al., 2011). Thus, the true domains of SHH expression are relatively ill defined. IHH is present during calvarial osteoblastic development and is expressed during osteoblast proliferation at the osteogenic fronts in mice, (the leading edge of the ossifying calvarial bone; Jacob et al., 2007). In another study, IHH expression was also found in the sagittal suture mesenchyme albeit to a lesser degree than in ossifying bones and osteogenic fronts in E17.5 mice (Lenton et al., 2011). Thus, the expression patterns of SHH and IHH differ in the cranial suture complex, although some discrepancies regarding the precise domains of SHH expression exist in the literature.

The domain-specific HH ligand expression has led investigators to examine different plausible functions for SHH and IHH ligands. The predominant presence of SHH in the suture mesenchyme and its overlapping expressing pattern with mesenchymal cell proliferation has led some to postulate that SHH has a role in maintaining suture patency (Kim et al., 1998). In fact, SHH may increase mesenchymal proliferation and suture mesenchyme thickness via promotion of MSX2, Muscle Segment Homeobox, a homeobox gene present in osteoblastic cells, in concert with BMP4 signaling (Alappat et al., 2003). Unlike other cytokines, which are concentrated in the dura mater of cranial sutures, SHH/IHH expression does not appear to be significantly derived from the underlying dura mater. In this way, HH signaling likely functions in a much different way than, for example, FGF2 or TGF-β (Mehrara et al., 1999; Moursi et al., 2002; Li et al., 2007; Gosain et al., 2009). In contrast, IHH is expressed by calvarial osteoblasts and disruption of IHH signaling impairs osteoblastogenesis (Murakami et al., 1997). IHH expression indicates that IHH induces PTCH and BMP2/4 expression, which results in intramembranous bone formation at the osteogenic fronts during intramembranous ossification and later suture fusion (Jacob et al., 2007; Lenton et al., 2011). Toward the completion of physiologic suture fusion, a decrease in osteoblast proliferation is observed, which coincides with a decrease in IHH expression. Another theory, although less frequently suggested, is that IHH functionally represses osteogenic lineage differentiation and that loss of IHH results in premature osteoprogenitor cell differentiation (Abzhanov et al., 2007). However, later studies by other research groups have not concurred with this observation (Lenton et al., 2011). Finally, IHH expression on the bony interfaces of synostotic rabbits may support the role of IHH in premature suture fusion (Nott et al., 2002a). Overall, the current available data suggests that SHH may prevent suture fusion and that IHH promotes calvarial ossification and sutural fusion. The idea that different, even opposing effects can result from two morphogens which use the same conserved pathway is a complex concept. Future research directly addressing this question may yield further insight.

Loss-of-function experiments involving either SHH or IHH results in profound craniofacial dysmorphisms. SHH^−/−^ animals die before or shortly after birth with a variety of developmental defects, including absence of distinct forelimb and hindlimb structures, and a dysostoic calvarial phenotype (McMahon et al., 2003). Unfortunately, dorsal midline structures fail to form and therefore null animals demonstrate secondarily impeded calvarial ossification (Chiang et al., 1996). In addition, it has been found that when SHH signaling is blocked in the brain but SHH is also applied early to the frontonasal ectodermal zone, the formation of the face and upper jaw is significantly improved. This suggests that the SHH signaling, which mediates brain to face interaction, is time-dependent (Chong et al., 2012). Approximately half of IHH^−/−^ mice die during mid-gestation due to yolk sac defects, while the remainder dies at birth, and is attributed to rib cage deformities and respiratory failure (Byrd et al., 2002). The IHH^−/−^ mouse demonstrates a dysostotic phenotype with reduced calvarial bone size and ossification, and grossly widened cranial sutures (St-Jacques et al., 1999; Razzaque et al., 2005; Kolpakova-Hart et al., 2008). However, unlike the SHH null mouse, the IHH null mouse cranial phenotype is not secondary to underlying CNS dysgenesis. Further studies demonstrated that reduced ossification in the IHH^−/−^ mouse is accompanied by global reduction of osteogenic markers and reduced BMP2/4 expression (Lenton et al., 2011). Overall, loss of function studies have suggested that IHH has a pro-osteogenic effect in calvarial ossification, while SHH studies are difficult to interpret due to widespread CNS malformations.

Both SHH and IHH ligands also interact with other signaling pathways in the developing skull, the most well studied of which are BMPs. BMPs, part of the TGF-β superfamily of growth factors, were first identified by their ability to induce ectopic ossification (Wozney et al., 1988). Currently, BMPs, such as BMP2, are in wide clinical use as a bone graft substitute for spinal fusion (Deyo et al., 2012; Hagen et al., 2012). The expression of the BMP signaling antagonist Noggin has been shown to be a critical regulator of cranial suture fusion (Warren et al., 2003; Jacob et al., 2007). In many studies, similarities were found between the patterning of SHH and BMP expression during neonatal craniofacial suture development (Liem et al., 2000; Santagati and Rijli, 2003) as well as with the transcription factor MSX2, a transcriptional repressor of neural crest-derived cells (Hodgkinson et al., 1993; Towler et al., 1994; Kim et al., 1998; Takahashi et al., 2001). However, the exact functional importance of SHH/BMP/MSX2 co-expression in the developing calvaria is not fully elucidated (Lallemand et al., 2009). Investigators have postulated that IHH also interacts with BMP signaling, and as previously mentioned, the IHH^−/−^ mouse demonstrates reduced calvarial BMP2/4 expression (Lenton et al., 2011). In addition, *in vitro* studies in suture mesenchymal cells found that IHH positively regulated BMP2 and BMP4 transcript abundance, associated with upregulation of osteogenic markers (Lenton et al., 2011). In sum, this data suggests that IHH lies upstream of BMP2/4 signaling and may be a part of the regulatory network that controls BMP expression, thereby influencing cranial ossification. In addition, and although not thoroughly investigated, HH signaling likely interacts with FGF signaling (including FGF-2 and FGF-9). FGF2/9 expression peaks during physiologic suture fusion (Fakhry et al., 2005), and activating mutations in FGFR-2 are the main cause of syndromic synostosis (McGillivray et al., 2005; Marie et al., 2008). The interactions between FGF and HH signaling have been more thoroughly studied in facial development, including the coordinated development of the medial nasal prominence (Hu et al., 2003). Studies suggest that retinoid signaling mediates FGF-8 and SHH expression and synchronizes the development of the face (Schneider et al., 2001). While coordinate effects of HH/FGF signaling certainly exist in cranial suture, a precise characterization of this link has yet to be determined. In summary, strong data exist to support regulation of BMP signaling by both SHH and IHH in the calvaria.

## Role of hedgehog signaling in endochondral processes

**Figure 3:**
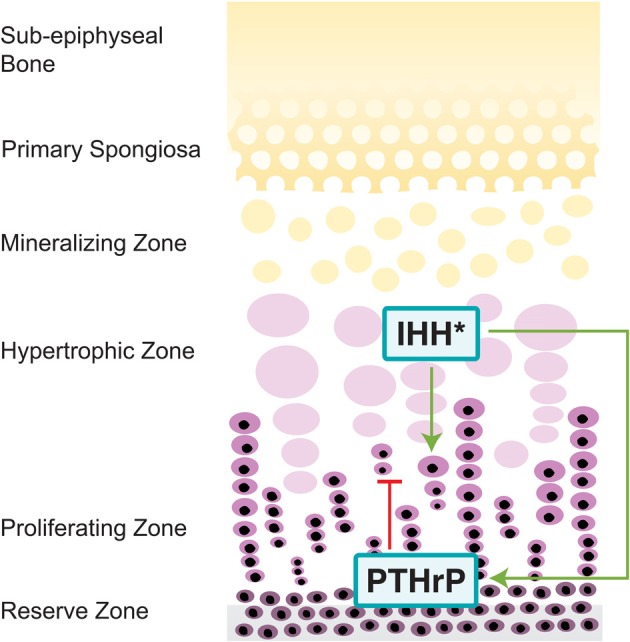
**Hedgehog pathway in the cranial base.** IHH, expressed in the pre-hypertrophic zones of growth plates, increases chondrocyte proliferation through increased expression of PTCH, a process mediated by intracellular component EVC (Long et al., 2001; Ruiz-Perez et al., 2007; Pacheco et al., 2012). Through a negative feedback loop, IHH also delays chondrocyte differentiation in order to sustain early chondrocyte production through synthesis of PTHrP, an IHH inhibitor (Lanske et al., 1996; Vortkamp et al., 1996; Kronenberg, 2003). ^*^IHH also regulates craniofacial morphogenesis through altering the IHH/PTHrP negative feedback loop, though it is primarily expressed in the spheno-occipital synchondroses (Tavella et al., 2004).

Another important aspect of HH signaling involves endochondral ossification, which is the process of bone development in the majority of bones in the axial and appendicular skeleton, as well as the cranial base (Nagayama et al., 2008). In contrast to intramembranous ossification (discussed previously), endochondral skeletogenesis occurs as mesenchymal cells differentiate into chondrocytes. As the cartilage enlarges, hypertrophic chondrocytes regulate matrix mineralization, vascularization and chondroclast attraction. As chondrocytes proliferate they are replaced by osteoblasts, which provide the scaffold for bone growth (Kronenberg, 2003). The cranial base is formed through endochondral ossification, in which the chondrocranium is formed and replaced by bones. These individual bones are connected by synchondroses, cartilaginous structures, which are similar to long bone growth plates (Nie, 2005). As with intramembranous bones, HH signaling is known to play an integral role in cranial base development (Figure 3).

SHH regulates craniofacial morphogenesis by altering the IHH/PTHrP negative feedback loop. Overexpression of SHH increases the amount of IHH inhibitor, PTHrP, thereby resulting in the failure of the cranial base to fully develop (Tavella et al., 2004). IHH works to delay the differentiation of chondrocytes through synthesis of PTHrP, which works on the PTH/PTHrP receptors in chondrocytes (Lanske et al., 1996). However, IHH is not present in the earliest stages of cranial base formation and patterning, as opposed to SHH, which indicates that their functions are not redundant (Nie et al., 2005). Increased SHH expression during later developmental period strongly correlates with IHH, and suggests that both IHH and SHH control subsequent bone formation in the cranial base. IHH, nonetheless, is the key regulator for endochondral ossification and is secreted by prehypertrophic chondrocytes and early hypertrophic chondrocytes (St-Jacques et al., 1999). IHH mainly functions to inhibit the progress of immature chondrocyte development in order to sustain early chondrocyte production (Vortkamp et al., 1996; Kronenberg, 2003). IHH in chondrocytes increases expression of PTCH, which then can activate SMO, allowing for increased chondrocyte proliferation (Long et al., 2001). Experiments with mice have shown that IHH regulates endochondral ossification in concert with a number of other proteins, including Ellis-van Creveld protein (EVC) and Kif3a (discussed below). IHH instigates the proliferation of chondrocytes and regulates maturation to promote cranial base elongation.

IHH is expressed in the pre-hypertrophic zones of growth plates while SHH expression is found in proximity to the spheno-occipital synchondroses. As the posterior portion of the cranial base is highly receptive to SHH, proper ossification occurs in both wild type and IHH^−/−^ mice. However, the anterior intrasphenoidal synchondrosis appears to be less susceptive to SHH, resulting in IHH^−/−^ mice with defective ossification in this area (Young et al., 2006). In addition IHH^−/−^ mice show ectopic calcification in the cartilaginous synchondroses between the basioccipital and exoccipital bones in the cranial base and delayed hypertrophic chondrocyte differentiation (St-Jacques et al., 1999). This delayed differentiation also results in IHH^−/−^ possessing disorganized synchrondosis growth plates and defects arise between IHH-producing pre-hypertrophic chondrocytes and PTHrP proliferating chondrocytes (Young et al., 2006). The many abnormalities produced from the IHH^−/−^ phenotype demonstrate the integral part that IHH plays within cranial base synchondrosis and chondrogenesis.

Additionally, mutations in proteins involved in IHH signaling also contribute to anomalies in the cranial base. EVC mediates signaling in the IHH pathway and is required for control over the rate of chondrocyte hypertrophy (Ruiz-Perez et al., 2007; Pacheco et al., 2012). EVC ^−/−^ mice exhibit morphological defects of the cranial base including midline gaps and partially fused basisphenoid suture in the neonatal stage (Pacheco et al., 2012). EVC ^−/−^ mice demonstrate a decreased expression in IHH downstream genes, such as PTCH and Gli1, suggesting that EVC is an intracellular component of the IHH signaling pathway. The motor protein Kif3a, as discussed earlier with its function intramembranous bone defects, also regulates the development of the cranial base. In Kif3a-deficient mice, there is a delay in chondrocyte maturation and hypertrophy, as IHH has an increased gradient of expression throughout the upper growth plate and IHH receptors, such as PTCH, are reduced (Koyama et al., 2007). Thus, IHH is required for synchondrosis organization and function, and disrupted regulation of IHH signaling by EVC or Kif3a results in significantly abnormal cranial base phenotypes.

## Unresolved concepts

While a number of studies have addressed the importance of HH signaling in cranial vault ossification and cranial suture biology, there are many questions that remain unanswered. Firstly, the precise expression pattern of SHH in the cranial suture is not yet agreed upon. Previous studies have found that SHH expression is principally observed in the ectodermal elements of the skull rather than in the bone or suture mesenchyme. In fact, using a reporter for HH activity, nearly complete loss of HH activity was observed in the skull and mesenchyme of IHH^−/−^ null animals (Lenton et al., 2011). This suggests that SHH may not be as relevant to calvarial ossification as other researchers suggest. However, through the use of *in situ* hybridization for SHH, expression was found on the sagittal and metopic sutures (Kim et al., 1998). This ambiguity could be the result of different timepoints of analysis (E16 vs. E18) or differences in experimental methodology. Secondly, there exist a large number of *in vitro* studies that demonstrate the basic pro-osteogenic effects of SHH (Yuasa et al., 2002; van der Horst et al., 2003; James et al., 2010, 2012; Tian et al., 2012). These studies, which have used diverse cell types including pre-osteoblastic cell lines and primary mesenchymal stem cell sources, would contradict the hypothesis that SHH functions to prevent suture ossification. In addition, a study on craniosynostotic rabbits documented increased expression of SHH in craniosynostotic sutures, (Nott et al., 2002b) and SHH overexpression, which simulates loss of PTCH function, has resulted in the absence of calvarial bones in the NBCCS mouse phenotype (Hu and Helms, 1999; Cobourne et al., 2009), further complicating the role of SHH in suture morphogenesis. In summary, the exact endogenous function of SHH on calvarial ossification has yet to be fully elucidated, either as an inhibitor or inducer of calvarial ossification/suture fusion. Thirdly, it remains uncertain if IHH and SHH in fact have redundant or even opposing functions in regulating calvarial ossification. Indeed as IHH and SHH function through the same cell surface receptor, it is difficult to determine a manner in which there would have antagonistic effects. A potential method to clarify the specific functions of Indian vs. Sonic may be through the use of bone specific knockout mice, allowing for examination of postnatal bone phenotypes in the absence of specific HH ligands.

## Conclusions

SHH and IHH have critical, sometimes synergistic, and currently debated biological roles in cranial suture development. Disruptions in either signaling ligand result in various defects in the development of cranial sutures. Examination of craniofacial defects arising from HH signaling abnormalities will provide new insights into the basic functions of the HH pathway in embryogenesis and patterning, and later bone and cartilage differentiation. Although much is known, there remain numerous questions for future study. Such questions include: (1) the precise function of SHH in cranial suture development, (2) whether IHH and SHH have different functions in the calvaria, and (3) improved understanding of the intersection of HH with other signaling pathways in craniofacial patterning, such as BMP signaling.

### Conflict of interest statement

The authors declare that the research was conducted in the absence of any commercial or financial relationships that could be construed as a potential conflict of interest.
